# Instant gait classification for hip osteoarthritis patients: a non-wearable sensor approach utilizing Pearson correlation, SMAPE, and GMM

**DOI:** 10.1007/s13534-024-00448-2

**Published:** 2025-01-09

**Authors:** Wiha Choi, Hieyong Jeong, Sehoon Oh, Tae-Du Jung

**Affiliations:** 1 Department of Robotics and Mechatronics Engineering, DGIST, Daegu, 711-785 Republic of Korea; 2 Department of Artificial Intelligence Convergence, Chonnam National University, 77 Yongbongro, Bukgu, Gwangju, 61186 Republic of Korea; 3 School of Medicine, Kyungpook National University Hospital, 680 Gukchaebosang-ro, Jung-gu, Daegu, 41404 Republic of Korea

**Keywords:** Gait assessment, Hip osteoarthritis, Pearson correlation coefficient, Symmetric mean absolute percentage error, Gaussian mixture model

## Abstract

This study aims to establish a methodology for classifying gait patterns in patients with hip osteoarthritis without the use of wearable sensors. Although patients with the same pathological condition may exhibit significantly different gait patterns, an accurate and efficient classification system is needed: one that reduces the effort and preparation time for both patients and clinicians, allowing gait analysis and classification without the need for cumbersome sensors like EMG or camera-based systems. The proposed methodology follows three key steps. First, ground reaction forces are measured in three directions-anterior–posterior, medial–lateral, and vertical-using a force plate during gait analysis. These force data are then evaluated through two approaches: trend similarity is assessed using the Pearson correlation coefficient, while scale similarity is measured with the Symmetric Mean Absolute Percentage Error (SMAPE), comparing results with healthy controls. Finally, Gaussian Mixture Models (GMM) are applied to cluster both healthy controls and patients, grouping the patients into distinct categories based on six quantified metrics derived from the correlation and SMAPE. Using the proposed methodology, 16 patients with hip osteoarthritis were successfully categorized into two distinct gait groups (Group 1 and Group 2). The gait patterns of these groups were further analyzed by comparing joint moments and angles in the lower limbs among healthy individuals and the classified patient groups. This study demonstrates that gait pattern classification can be reliably achieved using only force-plate data, offering a practical tool for personalized rehabilitation in hip osteoarthritis patients. By incorporating quantitative variables that capture both gait trends and scale, the methodology efficiently classifies patients with just 2–3 ms of natural walking. This minimizes the burden on patients while delivering a more accurate and realistic assessment. The proposed approach maintains a level of accuracy comparable to more complex methods, while being easier to implement and more accessible in clinical settings.

## Introduction

Hip osteoarthritis (OA) is a degenerative joint disorder characterized by the progressive degradation of cartilage in the hip joint. Approximately 4% of adults worldwide are diagnosed with hip OA, with prevalence rates increasing with age [1], resulting in roughly 10% of men and 18% of women over the age of 60 [2, 3]. Hip OA induces an abnormal gait pattern causing pain, stiffness, and restricted movement [4, 5]. Changes in gait greatly impact daily activities and reduce quality of life. Gait analysis provides valuable insights into disease progression and aids in creating personalized treatments to improve function. It is also crucial for evaluating treatment effectiveness and guiding rehabilitation. As a result, many studies on gait analysis in hip osteoarthritis patients [4–7].

Although all patients have the same disease, their gaits vary due to factors like disease severity, pain level, and reduced muscle mass [8–10]. These factors cause patients to adopt different gait strategies[11–14]. Therefore, categorizing a patient’s gait pattern is crucial for customized rehabilitation strategies. Many studies have highlighted that personalized rehabilitation through patient clustering yields significantly superior rehabilitation outcomes for OA patients compared to standardized rehabilitation [15–17]. For this reason, multiple studies are currently in progress to classify the patients based on their gait patterns [9, 18–20].

In order to apply this gait classification in clinical practice, two prerequisites must be met.

Firstly, it’s crucial to replicate conditions similar to their daily walking environment. However, external devices like EMG can alter natural gait and create unrealistic conditions. Attaching these devices, often requiring undressing, is inconvenient and adds unnecessary burden to the patient [21–23]. For this reason, analyzing gait without attaching equipment is preferable. Studies show that tools like force plates or insoles can effectively analyze gait, providing accurate measurements without the need for extra sensors [24–26]. Furthermore, extended walking during gait experiments can cause fatigue and affect natural gait patterns. To avoid this, we used a force plate for brief assessments, requiring only 2–3 ms of gait. This method reduces preparation time and patient burden, ensuring efficient and accurate gait analysis. Moreover, the data from the force plate comprehensively reflect the movements of both the upper and lower limbs, as well as the forces generated, providing intuitive and straightforward results [27, 28]. Since it is impractical to manually observe and evaluate the movements of all joints, assessing the final outcome (ground reaction force) based on force plate data is both convenient and feasible [29–32].

Secondly, it is essential to use quantitative indicators for assessing gait ability and classifying gait patterns [6, 7]. Gait ability is quantified by comparing the similarity between the gait pattern of healthy controls and patients. This similarity can be categorized into trend similarity and scale similarity. Trend similarity refers to the alignment of increasing or decreasing patterns in two time series, while scale similarity refers to the overall magnitude comparison. In gait analysis, both trend and scale similarity are critical for evaluating gait ability [33, 34]. Trend similarity can be measured using the Pearson correlation coefficient [35–38]. Scale similarity, on the other hand, can be measured using the Symmetric Mean Absolute Percentage Error (SMAPE). Both indicators provide standardized values [39, 40], making the interpretation of gait ability more intuitive and suitable for use as variables in gait classification [41, 42]. Therefore, this study employed the Pearson correlation coefficient for trend similarity and SMAPE for scale similarity.

Lastly, a soft clustering technique that forms flexible clusters should be utilized. Gait exhibits subtle variations even when the same individual walks under identical conditions [43–45]. Additionally, due to the numerous variables involved in classification and the overlap or similarity between gait patterns, clear distinctions are often difficult to make. Therefore, soft clustering techniques, which allow for the formation of flexible clusters, are well-suited for gait pattern classification. For these reasons, the Gaussian Mixture Model (GMM) technique were employed to cluster patient gait patterns [19, 46]. Advantages of GMM include its ability to model complex data distributions flexibly by using multiple Gaussian components, allowing for effective clustering.

In summary, utilizing only force plate data for gait assessment and classification reduces patient burden and enables a more natural evaluation of gait patterns. The application of the Pearson correlation coefficient and SMAPE as metrics allows for intuitive interpretation of results by capturing both trend and scale similarities. Moreover, gait analysis from both kinematic and kinetic perspectives (joint angles and moments) were performed on the classified patient groups, providing a foundation for distinguishing hip osteoarthritis (OA) patients. This study proposes a low-burden and efficient approach for classifying the gait patterns of hip OA patients using only force plate data. By integrating the Pearson correlation coefficient and SMAPE with a Gaussian Mixture Model (GMM), the proposed methodology offers a robust framework for immediate and effective gait pattern analysis.

## Materials and methods

### Whole procedure for proposed methodology for gait assessment and classification

**Fig. 1:**
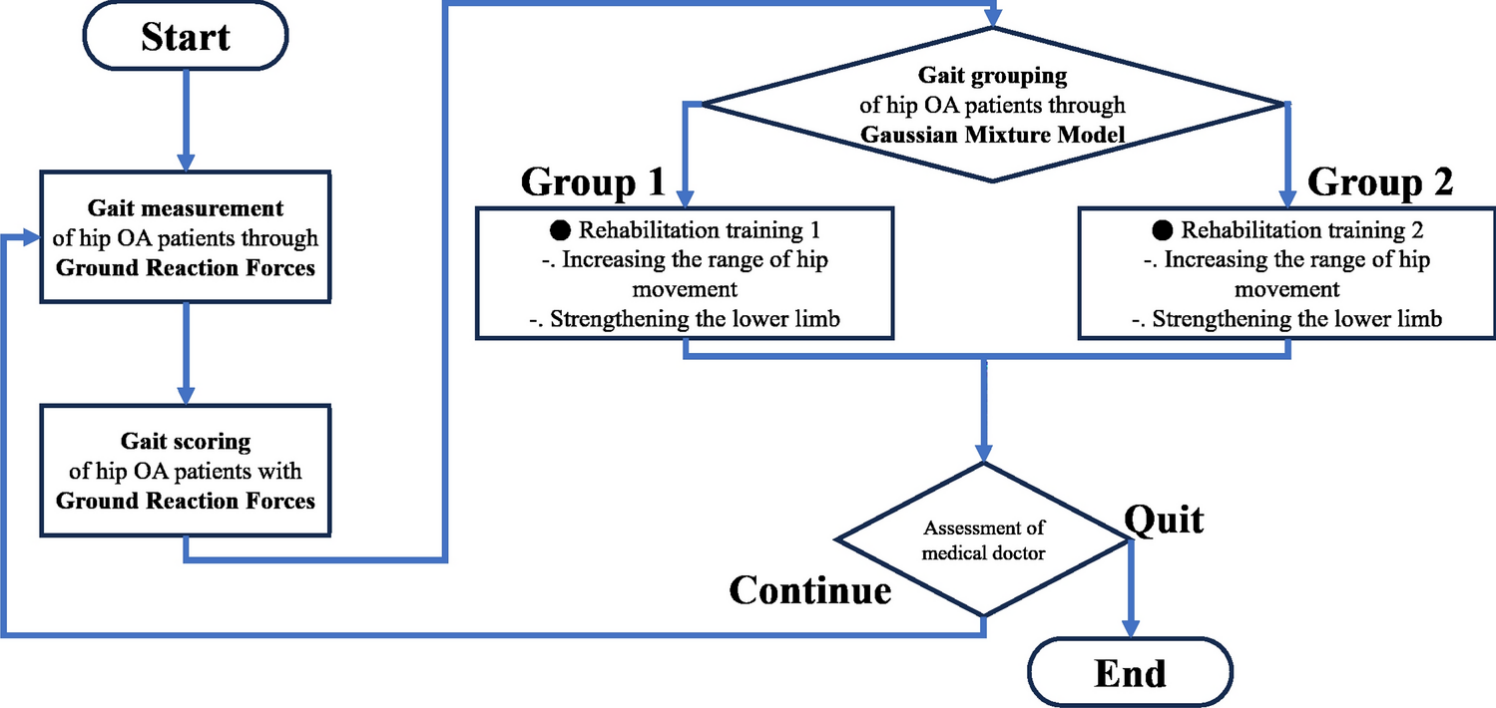
This flowchart illustrates the overall process introduced in this study

Figure 1 depicts the overall process of the gait assessment and gait pattern classification conducted in this study.

(i) Gait measurement is performed by walking 2–3 m on force-plate. Ground reaction force data of 3 axes axes (anterior–posterior, medial–lateral, vertical) are obtained.
(ii) The ground reaction force data obtained above are used to assess the gait ability of hip OA patients in comparison to healthy controls, employing the Pearson correlation coefficient and SMAPE, resulting in six values derived from each gait trial.
(iii) The obtained Pearson correlation coefficient and SMAPE values are utilized to classify patient gait patterns using GMM.
(iv) Based on the classified groups, the gait pattern of each patient is discerned, and customized Rehabilitation is implemented accordingly.

### Subject

Patients with hip osteoarthritis (OA) included in this study were those classified as grade 3 or 4 according to the Kellgren–Lawrence grading system [47]. All patients were candidates for total hip arthroplasty, and the grading was determined based on the clinical assessment of an orthopedic surgeon. Patients with hip OA graded as 0 to 2 were excluded from the study as they were not considered candidates for hip replacement surgery. Additionally, individuals with a history of other lower limb musculoskeletal disorders apart from hip OA were also excluded from the study [48]. All patients participate in gait experiments within 3 months of being diagnosed with hip OA through X-ray examination 16 patients with hip OA (9 women and 7 men, age: 56 ± 13 years, weight: 67 ± 11 kg, height: 164 ±6 cm) and 16 healthy subjects (8 women and 8 men, age: 56 ± 9 years, weight: 64 ± 10 kg, height: 163 ± 7 cm) who had no history of disorder related to gait have participated in gait experiments.

Prior to acquiring the measurements, experimental participants walked on the floor for 5 min to induce their natural gait. Subsequently, participants walked on the force-plates multiple times, and 3 trial datasets were selected, wherein participants accurately stepped on the force-plate.

All subjects voluntarily signed written consent forms for the experiment, approved by the Kyungpook National University Chilgok Hospital Institutional Review Board (IRB No.2018-05-008). This study followed the policy statement concerning the Declaration of Helsinki.

### Experimental system

**Fig. 2:**
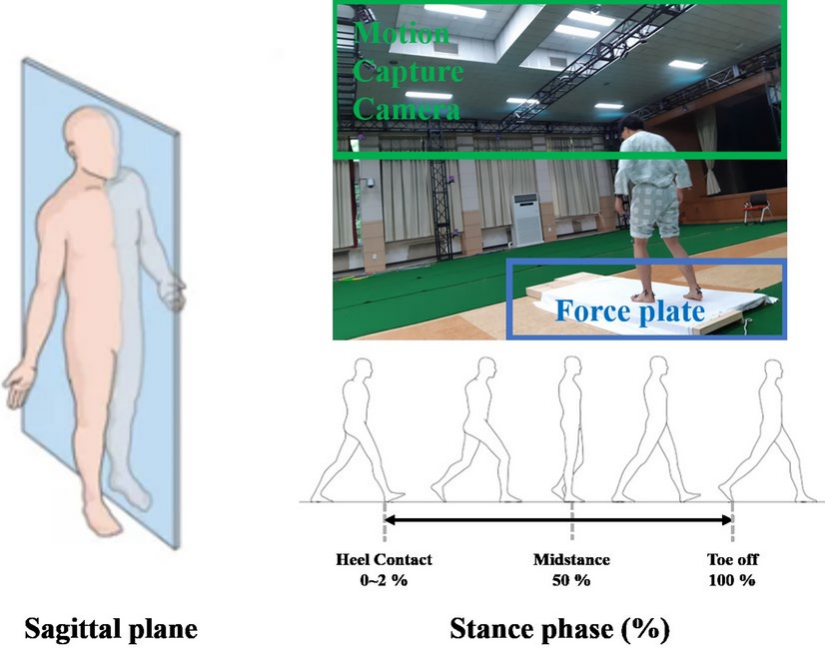
The figure in left illustrates the definition of sagittal plane. The figure in the upper right illustrates gait experiment environment with motion capture camera and force-plate. The picture in the bottom right depicts the stance phase as defined in this study

The experiments were performed using a Vicon motion capture camera (33EA, Vicon, Oxford, UK) and force-plate (2EA, AMTI, Boston, US) as seen in Fig. 2. The marker attachment was performed according to plug-in-gait (lower limb) marker sets.

During the gait experiment, data for the 3-axis Ground Reaction Force (GRF) is obtained from the force-plate in stance phase as seen in the lower right side of Fig. 2. Among these, the 3-axis GRF on the affected-side is acquired. Through the integration of Vicon camera and the force-plate, inverse dynamics calculations are performed using Plug-In Gait model [49–51]. This allows obtaining the angles and moments in the sagittal plane for hip, knee, and ankle joint as seen in the upper right side of Fig. 2.

Data analysis was carried out using Google Colab, and the Gaussian Mixture Model (GMM) implemented here utilized the GMM function from the scikit-learn package [52].

### Indices for assessing a patient’s gait ability

#### Pearson correlation coefficient for gait similarity in view of trend

Pearson correlation coefficient is an index that quantifies the similarity between 2 time-series data in view of trend [35, 36, 53]. The Pearson correlation coefficient formula is expressed as follows in Eq. (1):

1 $$\begin{aligned} R = \frac{\sum _{i=1}^{N}(H_i-\overline{H})(P_i-\overline{P})}{\sqrt{\sum _{i=1}^{N}(H_i-\overline{H})^{2}\sum _{i=1}^{N}(P_i-\overline{P})^{2}}} \end{aligned},$$

where *R* represents Pearson correlation coefficient and *N* represents the number of data points. $H_i$ represents the *i*th value of healthy subjects’ gait time series data, and $P_i$ represents the *i*th value of the patient’s gait time series data. $\overline{H}$ represents the average value of healthy subjects’ gait time series data, and $\overline{P}$ represents the average values of the patient’s gait time series data.

#### Symmetric mean absolute percentage error (SMAPE) for gait similarity in view of scale

The formula of Symmetric mean absolute percentage error (SMAPE) [39, 40] is as shown in Eq. (2).

2 $$\begin{aligned} SMAPE = \frac{1}{N}\sum _{i=1}^{N}\frac{|H_i-P_i|}{\frac{|H_i|+|P_i|}{2}} \end{aligned},$$

where *N* represents the number of data points. $H_i$ represents the *i*th value of healthy subjects’ gait time series data, and $P_i$ represents the *i*th value of the patient’s gait time series data. $\frac{|H_i|+|P_i|}{2}$ represents the average of the *i*th values of healthy subjects and patients. By dividing $|H_i-P_i|$ by this value, it helps resolve the scaling disparity issue and prevents potential division by zero problems [41, 54, 55].

SMAPE typically produces values between 0 and 1. values of SMAPE approaching zero indicates that gait pattern of patient of hip OA is similar with healthy controls. To provide an intuitive understanding of gait ability, an index known as the Symmetric Mean Absolute Percentage Error (SMAPE) score. The SMAPE score formula is expressed as follows in Eq. (3):

3 $$\begin{aligned} SMAPE\;score = 1- SMAPE \end{aligned}$$

### Classification of gait patterns in hip OA patients using Gaussian mixture model (GMM)

The 3 axis ground reaction force data (medial–lateral, anterior–posterior, vertical) measured on a force-plate are utilized for gait classification. To quantify the trend similarity of gait time series data, Pearson correlation coefficients are computed comparing with healthy control data. On the other hand, to quantify the scale similarity of gait time series data, the Symmetric Mean Absolute Percentage Error (SMAPE) are computed comparing with healthy control data. In summary, a total of six variables are used in Gaussian Mixture Model (GMM) clustering, incorporating both trend and scale similarities.

The mean values of the gait variables of healthy controls are standardized to 1. Subsequently, employing these values as references, comparisons and quantification of time-series data from healthy subjects and individual patient data are conducted using the Pearson correlation coefficient and Symmetric Mean Absolute Percentage Error (SMAPE).

GMM is a probabilistic model that represents data as a mixture of Gaussian distributions [43]. Employing the Expectation-Maximization (EM) algorithm, GMM estimates parameters such as means and covariance, providing a flexible framework for clustering and density estimation [45]. Its probabilistic nature and capacity to model complex data distributions make it widely applicable in various fields [19].

The probability density function of GMM is as follows.

4 $$\begin{aligned} p(x) = \sum _{k=1}^{K}\pi _{k}N(x|u_{k},\Sigma _{k}) \end{aligned}$$

where *K* represents the number of cluster as hyper parameter. *p*(*x*) represents the probability density function for each data point *x* in a GMM. $N(x|u_{k})$ represents multivariate Gaussian probability density function, number of data points. $\pi _{k}$ represents the mixing coefficient for cluster *k*. $\Sigma _{k}$ represents covariance matrix of cluster *k*.

### Variables for gait analysis of each group

To analyze the gait patterns of hip OA patients classified by GMM, the gait patterns of each group are compared from both kinetics and kinematics perspectives.

In the kinematics dimension, the angles of the hip, knee, and ankle joints in the sagittal plane is compared. In the kinetics dimension, the moments of the hip, knee, and ankle joints in the sagittal plane is compared.

## Result

### Results of trend and scale similarity in hip OA patients

Table 1 presents the trend similarity and scale similarity of the participants’ gait. All three axes of the GRF were compared, and the similarities were quantified. A value closer to 1 indicates greater similarity to the gait of healthy individuals. The high trend similarity (Pearson coefficient) implies that the gait pattern resembles that of healthy controls, while the high scale similarity (SMAPE) implies that a similar level of ground reaction forces are generated during gait as that of healthy controls. The values obtained were then used to classify the patients’ gait patterns using GMM.

**Table 1:** This table presents the trend similarity (Pearson correlation coefficient), scale similarity (SMAPE), clustering results using GMM, and the pre-experiment diagnosed Kellgren–Lawrence grade for all patients who participated in the experiment

Indice	Trend similarity (Pearson coefficient)			Scale similarity (SMAPE)			Clustering	Kellgren–Lawrence
Subject	GRF ML	GRF AP	GRF V	GRF ML	GRF AP	GRF V	Result using GMM	Grading
Subject 1	0.40	0.41	0.93	0.61	0.65	0.97	Group 1	3
Subject 2	0.35	0.44	0.93	0.61	0.64	0.94	Group 1	3
Subject 3	0.21	0.40	0.95	0.63	0.64	0.92	Group 1	3
Subject 4	0.18	0.25	0.96	0.64	0.63	0.91	Group 1	3
Subject 5	0.45	0.38	0.95	0.60	0.98	0.93	Group 2	4
Subject 6	0.73	0.57	0.93	0.55	0.70	0.94	Group 1	4
Subject 7	0.92	0.86	0.93	0.51	0.76	0.94	Group 2	4
Subject 8	0.87	0.89	0.93	0.51	0.73	0.93	Group 2	4
Subject 9	0.83	0.88	0.96	0.52	0.73	0.94	Group 2	4
Subject 10	0.80	0.88	0.97	0.52	0.69	0.93	Group 2	3
Subject 11	0.85	0.87	0.98	0.65	0.69	0.94	Group 2	4
Subject 12	0.89	0.88	0.96	0.78	0.71	0.95	Group 1	4
Subject 13	0.92	0.91	0.95	0.91	0.71	0.96	Group 1	4
Subject 14	0.89	0.92	0.95	0.78	0.74	0.92	Group 1	3
Subject 15	0.75	0.87	0.90	0.65	0.75	0.87	Group 1	3
Subject 16	0.73	0.86	0.91	0.53	0.78	0.83	Group 2	4

GRF ML refers to the medial–lateral ground reaction force, GRF AP refers to the anterior–posterior ground reaction force, and GRF V refers to the vertical ground reaction force. The values shown are the averages of the three experimental trials. The participant numbers correspond to the order in which the experiments were conducted

#### Number of clustering in Gaussian mixture model

Bayes Information Criterion (BIC) techniques to determine the optimal number of groups for GMM are employed [56, 57]. The BIC results indicate that the optimal clustering group number is 2. Therefore hip OA patients were clustered into 2 groups. Additionally, in the conducted K-means clustering [58], the optimal number of clusters was determined using both the elbow method and silhouette score [59, 60]. The results obtained were consistent with those from the aforementioned analyses.

#### Clustering result using GMM

Clustering was performed on the data from 3 trials for each patient, and the results revealed that the trial data for each patient were consistently classified into the same group (intra-validation).

Group 1 and Group 2 are distinguishable, with 9 patients assigned to Group 1 and 7 to Group 2 as seen in Table 1. In addition, k-means clustering analysis, consistent clustering results were obtained. Furthermore, by supplementing the original 3 axis ground reaction forces with the anterior–posterior trajectory of the Center of Pressure (COP) and applying GMM and K-means clustering, the clustering results aligned with the original outcomes.

#### Comparison of gait characteristics among each group

Significant differences in gait variables among healthy control, and hip OA patient groups are observed in hip angle, hip moment, and knee moment in the sagittal plane. The knee angle, ankle moment, and ankle angle in the sagittal plane do not exhibit significant differences among the healthy controls and hip OA patient groups.

**Fig. 3:**
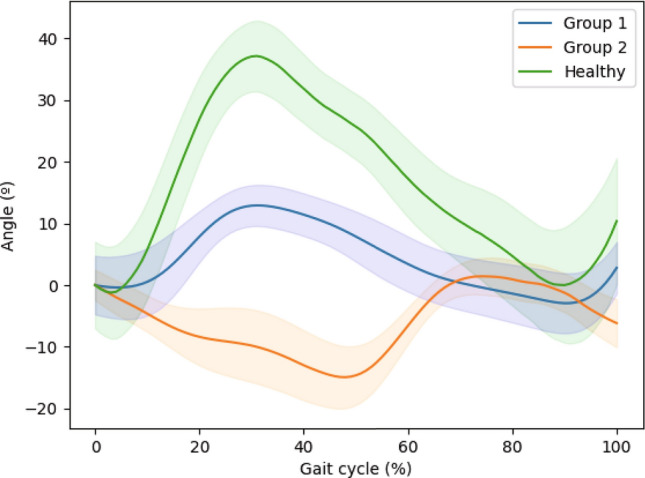
Hip angles were measured in sagittal plane across the gait cycle for healthy controls and hip osteoarthritis (OA) patients in Group 1 and Group 2. The green line represents the mean values for healthy controls, the blue line represents the mean values for Group 1, and the orange line represents the mean values for Group 2. 95% confidence intervals are included

Figure 3 illustrates the sagittal plane hip angles across the gait cycle for healthy controls and hip OA patients in Group 1 and hip OA patients in Group 2.

**Fig. 4:**
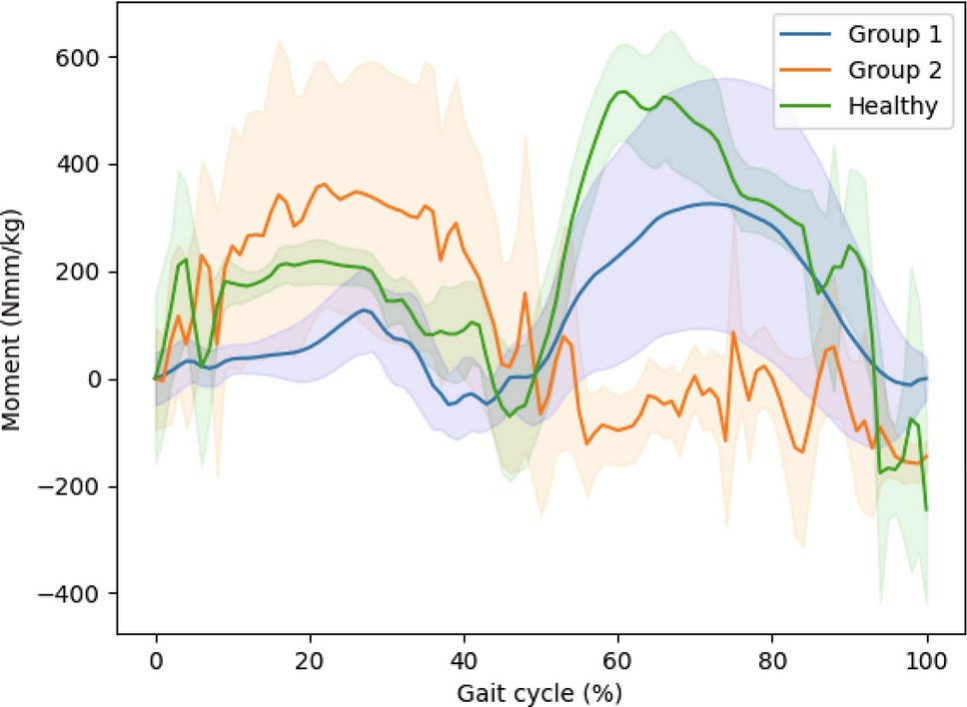
Hip moment were measured in sagittal plane across the gait cycle for healthy controls and hip osteoarthritis (OA) patients in Group 1 and Group 2. The green line represents the mean values for healthy controls, the blue line represents the mean values for Group 1, and the orange line represents the mean values for Group 2. 95% confidence intervals are included

Figure 4 illustrates the sagittal plane hip moments across the gait cycle for healthy controls and hip osteoarthritis (OA) patients in Group 1 and hip OA patients in Group 2.

**Fig. 5:**
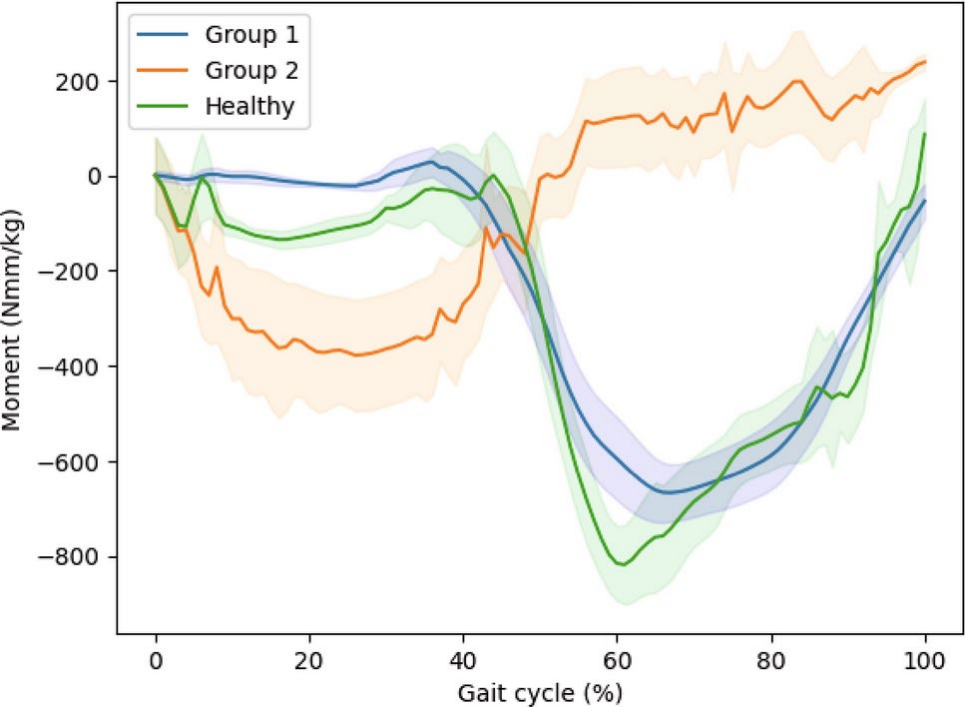
Knee moment were measured in sagittal plane across the gait cycle for healthy controls and hip osteoarthritis (OA) patients in Group 1 and Group 2. The green line represents the mean values for healthy controls, the blue line represents the mean values for Group 1, and the orange line represents the mean values for Group 2. 95% confidence intervals are included

Figure 5 illustrates the sagittal plane knee moments across the gait cycle for healthy controls and hip osteoarthritis (OA) patients in Group 1 and hip OA patients in Group 2.

## Discussion

### Contribution points of proposed methodology

Several studies have meticulously examined and classified the gait characteristics of OA patients, focusing on the kinematic properties of the hip joint. These studies demonstrate significant strengths by enabling precise analysis and classification through the use of various sensors, which capture changes in hip joint angles as well as multiple gait variables such as cadence and stride length [61–63].

Nonetheless, this methodology places a substantial burden on the patient. Patients must visit facilities equipped with experimental apparatus such as motion capture markers, change into specialized attire for the experiment, and undergo the attachment of sensors. Additionally, the experimenter is required to set up the experimental environment, attach sensors, and possess the necessary expertise for accurate execution. This process results in significant inconvenience and requires considerable time and effort from both the patient and the experimenter. In this study, a method to intuitively quantify and classify the gait ability and pattern of patients with hip OA in terms of trend and scale solely based on just 2–3 m gait is proposed [64].

In addition to the convenience of classification experiments, the proposed methodology presents another significant advantage in its ability to reflect the weight-shifting patterns of OA patients. Hip OA patients often exhibit excessive or inefficient weight shifting during gait as a result of muscle atrophy and fear of walking. Although the information obtained through the force plate is more limited compared to traditional methods, it nonetheless enables feasible gait assessment and classification. In this regard, evaluating gait ability using only force plate data offers both efficiency and effectiveness. Many studies have been conducted that classify gait and movement using only force plate data [29–32, 65, 66]. On the other hand, by using the Pearson correlation coefficient and SMAPE to measure both the trend and scale similarity of gait, this method can help determine whether a patient’s walking issues are due to an abnormal gait pattern or an inability to exert sufficient force. Since many hip OA patients struggle to generate adequate force, SMAPE is particularly useful in assessing gait similarity for patients with reduced strength. Both metrics provide a clear and straightforward interpretation of the patient’s gait condition.

Through this method, it is revealed that the gait patterns of patients with hip OA can be distinguished into 2 patterns. The differences between these groups are found in the sagittal plane’s hip and knee moments, as well as hip angle, suggesting the potential for personalized rehabilitation strategies.

### Gait pattern difference between hip OA groups

In the sagittal plane, significant differences are observed among groups in hip angle, as well as moments at the hip and knee.

It can be noted that significant differences occur in both the angle and moment at the hip joint affected by the osteoarthritis, and compensatory actions in response to this are evident in the knee moment. However, no significant differences are observed in the angle and moment of the ankle, relative to other joints, which suggests a need to focus on hip and knee joints in rehabilitation.

Through this, it was evident that the key differences in distinguishing between the healthy controls and patients with hip OA, as well as within hip OA patients, lie in the sagittal plane’s hip moment, knee moment, and hip angle.

In the case of the hip angle, flexion occurs during the early phase of gait, transitioning to extension in the mid-to-late phase, resulting in a natural gait pattern. As the hip angle undergoes flexion, the hip moment is generated to contribute to the deceleration of gait, while during the extension phase of the hip angle, the hip moment is generated to facilitate the acceleration of gait [67].

In contrast, Group 1 exhibits hip angle movements similar to those of the healthy control. In other words, flexion and extension occur in a manner consistent with typical gait patterns of healthy control. However, the range of motion (ROM) is relatively limited. The hip moment generates at the appropriate instance for deceleration and acceleration in gait, yet it does not manifest with a sufficient magnitude. Patients in Group 1 can achieve improved gait ability through targeted training to increase hip range of motion and strengthen the lower limb for enhancing hip moments [68–70].

In the case of Group 2, the movement of the hip angle exhibits a form markedly different from typical gait. The hip angle does not follow a sequential pattern of flexion and extension; rather, there is a slight initial occurrence of extension, with minimal subsequent flexion. This is presumed to result from a defensive gait pattern, characterized by exerting excessive force on the hip joint, as a response to pain and fear associated with gait. As evidence, it can be observed that the hip moment utilized for deceleration is stronger than that in the healthy control. Additionally, the excessive force applied to the lower limbs results in an exaggerated effect of the knee moment during deceleration. However, neither the hip moment nor the knee moment is effectively utilized during acceleration. Therefore, for patients in Group 2, rehabilitation efforts should focus on gait training that emphasizes familiarity with gait patterns and addresses the fear of gait, rather than strengthening exercises [4, 8].

Both participants with Kellgren–Lawrence (KL) grades 3 and 4 were included in the experiment, and the classification results divided their gait patterns into Group 1 and Group 2. The Kellgren–Lawrence grade is a measure of the severity of hip OA, with grade 4 representing more advanced hip OA. Given that Group 2 displayed gait patterns that deviated significantly from those of healthy individuals, it would be expected that KL grade 4 would correspond to Group 2. However, the association between KL grade 4 and Group 2 was only 68.5%, indicating a relatively low correlation. The discrepancy between expert opinions based on X-rays and the gait classification results suggests that the deterioration in gait patterns may not necessarily be solely attributed to the severity of hip OA. Psychological factors, such as the subject’s fear of walking or their level of engagement during the assessment, may also have influenced the gait classification.

### limitation and future work

The current number of participants is not sufficient to represent the gait patterns of hip OA patients in KL grades 3 to 4. Additional experiments will be conducted to explore whether there are more diverse gait pattern groups among hip OA patients and to analyze the gait patterns of those groups. Although the current sample size is limited, as more participants are included and data accumulates, this methodology has the potential to be established as a convenient and rapid tool for gait assessment and rehabilitation planning for patients.

## Conclusion

This paper presents a methodology for assessing gait ability and classifying gait patterns of hip OA patients using only data from their daily walking. This approach allows for easy and low-burden evaluation and classification of gait in hip patients, and it suggests the potential for personalized rehabilitation strategies.
